# Strategic Paragraphing 2.0: Techniques for Enhancing Inter-Paragraph Coherence

**DOI:** 10.5334/pme.727

**Published:** 2023-01-06

**Authors:** Yang Yann Foo, Lorelei Lingard

**Affiliations:** 1 Centre for Healthcare Education Research & Scholarship, and Department of Technology Enhanced Learning and Innovation, Office of Education, Duke-NUS Medical School, Singapore; 2 Centre for Education Research & Innovation, and Department of Medicine, Schulich School of Medicine & Dentistry and Faculty of Education, Western University, London, Canada

## Abstract

Most of us pay attention to intra-paragraph coherence: the idea that the sentences within a paragraph should logically develop a single idea. But we forget, or we struggle to master inter-paragraph coherence: the idea that paragraphs should be arranged so that our argument develops logically. Proximity isn’t always enough to signal to readers how paragraphs are building on one another to create a compelling argument. Readers may still end up having to infer how one paragraph is linked to the next, and when they can’t make those inferences (or they make them incorrectly) coherence suffers. This Writer’s Craft offers techniques to enhance inter-paragraph coherence more consciously so that readers don’t fall through the cracks between our paragraphs.

A seismic tremor rocked my writing world this year when I received personalized feedback on my draft during a writing workshop. The margin comment said, “You tend not to wrap paragraphs – which is OK, as long as the next topic sentence backward signals and/or the relationship is self-evident”. I wasn’t aware that my paragraphs were missing something essential (what, I wondered, is a “wrap”?), nor had I thought much about how readers get from one paragraph to the next. I just tended to put related paragraphs in close proximity to each other. Done, right? Wrong.

Sending the right signals to help readers follow our argument is something we owe to ourselves as writers: after all, we move mountains to get published, so it’d be most dispiriting if our papers languish in the valley of the unread. All else being equal, readers will gravitate towards writing that requires less effort to process – that has better readability. One important aspect of readability is paragraph coherence. Most of us pay attention to intra-paragraph coherence: the idea that the sentences within a paragraph should logically develop a single idea [1,2]. This aspect of coherence received attention in an earlier Writer’s Craft [2]. But we forget, or we struggle to master *inter*-paragraph coherence: the idea that paragraphs should be arranged so that our argument develops logically. Proximity isn’t always enough to signal to readers how paragraphs are building on one another to create a compelling argument. Readers may still end up having to infer how one paragraph is linked to the next, and when they can’t make those inferences (or they make them incorrectly) coherence suffers. This Writer’s Craft offers techniques to enhance *inter*-paragraph coherence more consciously so that readers don’t fall through the cracks between our paragraphs.

## Techniques to effect inter-paragraph coherence

A technique to establish inter-paragraph coherence is to chain-link information to create “a continuous flow of thought” for readers [3, p.230]. Readers will discern this continuity when writers follow a linguistic principle called “Given-New” where they express known information (the “Given”) before providing unknown information (the “New”) [4]. The Given-New principle helps to fulfil readers’ expectations when they read [5]: when a paragraph ends, they leave with a sense of what to carry forward, and when a new paragraph begins, they expect a backward connection to what came immediately before.

Writers can link Given-New information across paragraphs using three techniques: forward signaling, backward signaling, and lexical cohesion. We will work through a series of examples, illustrating how these techniques enhance coherence.

### Forward Signaling

Forward signaling can affect inter-paragraph coherence through the chain-linking of topic and wrap sentences [2,6,7]. Topic and wrap sentences are located at the beginning and end of a paragraph. A topic sentence announces the main idea while a *wrap* sentence reinforces that main point and forward signals what is to come in the subsequent paragraph:

Interprofessional collaboration is widely considered to be crucial for patient safety but fostering collaborative practice has largely remained an elusive goal. Physician resistance, in particular, has remained strong, which is not unexpected as medical doctors have historically been dominant in healthcare. Even with interventions from leadership, older non-collaborative practices have persisted. This stasis exists despite countless attempts to understand collaboration challenges by drawing on theories from psychology and sociology. *To break this stasis, a previously untapped but viable alternative has been proposed – systems theories*.

What does the reader take with them as they leave this paragraph? Without a *wrap*, they might select any of the details provided. Perhaps they’ll settle on physician resistance or the failure of research efforts: if the writer is lucky they’ll guess correctly. But coherence shouldn’t depend on luck. The *wrap* directs the reader to focus on the failure of research efforts. Then, having reinforced this as the main idea of the paragraph, the *wrap* can then forward signal what is to come – how systems theories could break the stasis.

The forward-signal only works to create a chain-link when the topic sentence of the next paragraph fulfils what was promised in the *wrap*. This would be the case if the next paragraph began with a topic sentence like: “Systems theories, with their ability to identify barriers that exist in micro-, meso-, exo- and macro-systems, would allow researchers to examine collaboration challenges in a comprehensive yet integrated manner”.

However, the chain-link is not realized if the next paragraph opens with a sentence like: “Psychological and sociological theories have informed a large body of interprofessional collaboration work.” The reader is expecting the New information at the start of paragraph 2 to expand on the Given information at the end of paragraph 1: what “Systems theories” can do to break the research stasis. If that’s not how paragraph 2 opens, coherence can suffer.

In sum, a wrap sentence reinforces Given information and also forward signals how that information is linked to New information in the following paragraph. When the next topic sentence repeats that New information, the continuity created by this chain-linking helps readers to discern clearly how the paragraphs are building on one another in service of an argument.

### Backward Signaling

Sometimes forward signaling isn’t ideal. Perhaps your paragraph has been long and complicated, and you’re loathe to add a new idea at the end. In such cases, backward signaling is another technique for inter-paragraph coherence. Backward signaling means that the topic sentence of the new paragraph begins with Given material from the preceding wrap sentence before going on to introduce New material. Below we revisit the preceding example to illustrate *backward signaling*:

Interprofessional collaboration is widely considered to be crucial for patient safety but fostering collaborative practice has largely remained an elusive goal. Physician resistance, in particular, has remained strong, which is not unexpected as medical doctors have historically been dominant in healthcare. Even with interventions from leadership, older non-collaborative practices have persisted. This stasis exists despite countless attempts to understand collaboration challenges by drawing on theories from psychology and sociology.

*To break this stasis*, researchers can use system theories to examine collaboration challenges in a comprehensive yet integrated manner.

In this example, the idea of Systems theories is not introduced at the end of the first paragraph. But the chain link is still formed by restating the Given (“this stasis”) as the new topic sentence opens. If this backward signal was left off and the next paragraph started with just “Researchers can use Systems theories to examine collaboration challenges…”, the reader could wonder, “Wait a second, what does Systems theories have to do with anything?” The backward signal makes that connection explicit, rather than leaving the reader to infer it.

Grammatically, backward signaling is achieved through the use of infinitive phrases, prepositional phrases, or conjunctive adverbs at the start of the new topic sentence [8]. Infinitive phrases are short fragments beginning with an infinitive verb: e.g., “To break this stasis”. Prepositional phrases are fragments beginning with prepositions, such as “For the purpose of breaking this stasis, researchers can use Systems theories”. Conjunctive adverbs do the work of transitioning between clauses and show relationships such as addition, clarification, comparison, contrast, emphasis, summary: e.g., “However, researchers can break the stasis by using Systems theories…”.

### Lexical Cohesion

The third technique for inter-paragraph coherence is lexical cohesion, which refers to the use of ***consistent terms*** to achieve continuity in meaning [9]. Often writers have a few key terms they use repeatedly in a paper, and they may be tempted to spice up their prose by using synonyms. Beware though: variety for variety’s sake can threaten coherence, making the reader work even harder to establish how paragraphs are building to create an argument. The following illustrates the important role that lexical cohesion plays when writers are linking Given-New information to create inter-paragraph coherence:

Interprofessional ***collaboration*** is widely considered to be crucial for patient safety. However, fostering ***collaborative*** practice has largely remained an elusive goal in the last three decades. This stasis exists despite countless attempts to understand collaboration challenges by drawing on ***theories*** from psychology and sociology.

To break the stasis, system ***theories*** can offer a more effective way of examining ***collaboration*** challenges in a comprehensive yet integrated manner.

Here, the consistent use of the terms “interprofessional ***collaboration***”, “***collaborative*** practice”, “***collaboration*** challenges” and “***theories***” links paragraph 2 to paragraph 1. The connectedness between the paragraphs would have been diluted had the writer interchangeably used ‘interprofessional communication/education/practice/care’ or ‘system concepts/system-based approaches’. Instead of focusing on the argument, readers might instead be forced to keep track of whether the different terms used are synonymous in meaning. Such distractions undermine the explicit chain-linking between the two paragraphs.

## How often and when to chain-link information

So, chain-linking can enhance readability, but *how often* should this technique be applied? Remember that it’s only part of a suite of coherence techniques that’s available to writers. For instance, writers may link Given-New information to a lesser degree when using headings and sub-headings. Consider the example below [10]:

The author and the journal’s editor need different things from your review, and so the risk that someone will be disappointed—or disheartened—is high. The author hopes for feedback that will strengthen their scholarship, while the journal wants an assessment of the paper’s quality and suitability for publication. How can we reconcile the sometimes competing purposes of our reviews to ensure they are meaningful? Let’s consider some helpful writing strategies.

Your conversation with the author:

Treat the feedback-forward parts of the review as a conversation, writing as though you were sitting down with the author(s) to talk about their work. The second person, used rarely in academic writing, works well here to build rapport and create a sense of intimacy.

Here, the writers did not chain-link the “helpful writing strategies” from the Wrap of the first paragraph to the Topic sentence of the second, which begins with a new idea “feedback-forward”. However, their use of the subheading “Your conversation with the author” suggests that this is the first of the writing strategies that were promised, keeping inter-paragraph coherence alive.

Another instance where chain-linking might not be needed to effect inter-paragraph coherence is when a short transition paragraph is being used to link two sections of a paper. Here’s an example: “Given the importance of these factors shaping resident shame, it is imperative to examine their implications for asking for help. In the next section, we will connect the help-seeking and shame literatures.” Following the transition paragraph, writers may use either a heading or sub-heading to begin a new section. However, for writers struggling with word limit, Given-New chain-linking can be more economical than transition paragraphs for ensuring inter-paragraph coherence. With no hard and fast rules for dictating how often writers should use a particular technique to effect coherence, such decisions should be made based on the specific writing context.

What about the question of *when* information should be chain-linked? Should writers do so as they’re writing, or should they chain-link after they’ve written the draft? The answer depends what kind of writers they are. For those who develop outlines before writing, it might help to incorporate chain-linking techniques from the start. But for those who prefer to fill pages as words and ideas form organically, stopping to chain-link might break their train of thought or even cause writer’s block. It may be better for such writers to leave chain-linking to the revision stage.

## Conclusion

Readers are unpredictable creatures, so don’t leave it to them to infer how your paragraphs connect. Inference is an effortful and unruly process, and readers may make wrong guesses about connections, undermining your developing argument. Use chain-linking of paragraphs so that readers can easily follow your arguments, and give your papers a chance of prospering in the land of the much-read.
